# The anti-tumor effects of AZD4547 on ovarian cancer cells: differential responses based on c-Met and FGF19/FGFR4 expression

**DOI:** 10.1186/s12935-024-03235-2

**Published:** 2024-01-25

**Authors:** Yoo-Young Lee, Ji-Yoon Ryu, Young-Jae Cho, Ju-Yeon Choi, Jung-Joo Choi, Chel Hun Choi, Jason K. Sa, Jae Ryoung Hwang, Jeong-Won Lee

**Affiliations:** 1 Division of Gynecologic Oncology, Department of Obstetrics and Gynecology, Samsung Medical Center, Sungkyunkwan University School of Medicine, 81 Irwon-ro, Gangnam-gu, Seoul, 06351 Korea; 2 Research Institute for Future Medicine, Samsung Medical Center, Sungkyunkwan University School of Medicine, 81 Irwon-ro, Gangnam-gu, Seoul, 06351 Korea; 3 Department of Biomedical Sciences, Korea University College of Medicine, Seoul, Korea; 4 Samsung Advanced Institute for Health Sciences and Technology, Sungkyunkwan University School of Medicine, Seoul, Korea

**Keywords:** Epithelial ovarian cancer, AZD4547, c-Met, FGF19, FGFR4, Patient-derived xenograft

## Abstract

**Background:**

The FGF/FGFR signaling pathway plays a critical role in human cancers. We analyzed the anti-tumor effect of AZD4547, an inhibitor targeting the FGF/FGFR pathway, in epithelial ovarian cancer (EOC) and strategies on overcoming AZD4547 resistance.

**Methods:**

The effect of AZD4547 on cell viability/migration was evaluated and in vivo experiments in intraperitoneal xenografts using EOC cells and a patient-derived xenograft (PDX) model were performed. The effect of the combination of AZD4547 with SU11274, a c-Met-specific inhibitor, FGF19-specific siRNA, or an FGFR4 inhibitor was evaluated by MTT assay.

**Results:**

AZD4547 significantly decreased cell survival and migration in drug-sensitive EOC cells but not drug-resistant cells. AZD4547 significantly decreased tumor weight in xenograft models of drug-sensitive A2780 and SKOV3ip1 cells and in a PDX with drug sensitivity but not in models with drug-resistant A2780-CP20 and SKOV3-TR cells. Furthermore, c-Met expression was high in SKOV3-TR and HeyA8-MDR cells, and co-administration of SU11274 and AZD4547 synergistically induced cell death. In addition, expressions of FGF19 and FGFR4 were high in A2780-CP20 cells. Combining AZD4547 with FGF19 siRNA or with a selective FGFR4 inhibitor led to significantly reduced cell proliferation in A2780-CP20 cells.

**Conclusions:**

This study showed that AZD4547 has significant anti-cancer effects in drug-sensitive cells and PDX models but not in drug-resistant EOC cells. In drug-resistant cells, the expression level of c-Met or FGF19/FGFR4 may be a predictive biomarker for AZD4547 treatment response, and a combination strategy of drugs targeting c-Met or FGF19/FGFR4 together with AZD4547 may be an effective therapeutic strategy for EOC.

**Supplementary Information:**

The online version contains supplementary material available at 10.1186/s12935-024-03235-2.

## Background

Epithelial ovarian cancer (EOC) is a gynecologic cancer with a high incidence [1] of an estimated 239,000 new cases annually worldwide and the highest mortality rate among gynecologic cancers [2]. With our growing understanding of cancer genetics and identification of specific targeted agents, there have been remarkable advances in treatment outcomes in some cancers including EOC. For example, maintenance therapy with poly-ADP ribose polymerase (PARP) inhibitors exhibits significant benefits on the survival of ovarian cancer patients, particularly those with mutations in *BRCA* or genes related to homologous recombination [3], which are present in approximately 20–50% of ovarian cancer patients [4]. Thus, there is a need to investigate new targets for EOC patients without these mutations, as they account for the majority of EOC cases.

Fibroblast growth factor receptors (FGFRs), which belong to the family of tyrosine kinase receptors, bind to fibroblast growth factors (FGFs). The FGF/FGFR signaling pathway has a critical role in both physiological and pathological processes in humans [5]. For example, the FGF/FGFR axis is involved in malignant transformation, tumor proliferation, metastasis, and chemo-resistance [6, 7]. FGFR aberrations were found in 7.1% of solid malignancies, and *FGFR1* amplification was most common (3.5%). In EOCs, these mutations were reported in up to 9% of cases, with the majority being FGFR1 amplifications, accounting for up to 5% [8]. Abnormal upregulation of FGFs (e.g., FGF1 and FGF3) has also been found in EOCs and stroma cells [9–11]; this can activate the FGF/FGFR axis through autocrine and paracrine mechanisms. Genetic aberration of FGFR genes such as amplification, point mutations, and gene fusion has been found in various cancers including breast, gastric, bladder, and non-small cell lung cancers [12]. Researchers and pharmaceutical companies have developed non-selective inhibitors targeting FGF/FGFR such as ponatinib, nintedanib, and dovitinib. Selective inhibitors including AZD4547, FGF401, LY2874455, and derazantinib were later developed and are being explored in preclinical and clinical trials. Na et al. reported that AZD4547 inhibited EOC growth [13]. However, there is a scarcity of studies investigating the role of FGF/FGFR axis blockade in EOC, particularly in terms of using selective FGFR inhibitors.

This study investigated the efficacy of targeting the FGF/FGFR pathway in EOC models. We showed significant anti-tumor effects of FGFR1–3 inhibition with AZD4547 in EOC models. These effects were not observed in drug-resistant cells highly expressing c-Met or FGF19/FGFR4, which could be treated with either c-Met-specific inhibitor, FGF inhibition, or an FGFR4 inhibitor combination. Therefore, we suggest that c-Met and FGF19/FGFR4 expression levels can potentially act not only as a predictive biomarker for AZD4547 treatment, but also serve as therapeutic targets for AZD4547-resistant EOC.

## Methods

### Cell lines and chemicals

Human EOC cell lines (SKOV3ip1 [RRID:CVCL_0C84], HeyA8 [RRID:CVCL_8878], A2780-CP20 [RRID:CVCL_A5PS], HeyA8-MDR [RRID:CVCL_8879] and SKOV3-TR [RRID:CVCL_HF69]) were a gift from Dr. Anil K. Sood in the Department of Cancer Biology, University of Texas M.D. Anderson Cancer Center, TX, USA. A2780 was purchased from the European Collection of Cell Cultures (ECACC, Cat No.93,112,519, RRID: CVCL_0134). EOC cells were maintained in RPMI 1640 supplemented with 10% fetal bovine serum (FBS) and incubated in 5% CO_2_ at 37 °C. All experiments were performed with mycoplasma-free cells.

The selective FGFR1-3 inhibitor AZD4547, (N-[5-[2-(3,5-dimethoxyphenyl)ethyl]-2 H-pyrazol-3-yl]-4-(3,5-diemthylpiperazin-1-yl) benzamide) (AstraZeneca), the selective potent FGFR4 inhibitor FGF401 (roblitinib) (MedChemExpress), c-Met inhibitor SU11274 (SelleckChem) were resuspended in dimethyl sulfoxide (DMSO) at a concentration of 10 mmol/ml and diluted in culture media.

### Small interfering RNA (siRNA) transfection

FGF19-specific siRNA and negative control siRNA were obtained from Bioneer (Daejeon, Korea). The sense sequence for FGF19-specific siRNA was 5’-GUGCUUUCGAGGAGGAGAU-3’. Cells were seeded at a concentration of 2 × 10^5^ cells/well in 6-well plates in RPMI1640 with 10% FBS. siRNA was transfected into cells using Lipofectamine 2000 (Invitrogen, San Diego, CA, USA) in accordance with the manufacturer’s protocol.

### Western blot analysis

Cells were lysed using PRO-PRE Protein Extraction Solution (Intron Biotechnology, Seongnam, Korea), and protein concentration was determined using a Bradford assay kit (BIO-RAD, Hercules, CA, USA). Cell lysates (40 µg) were separated on 8% or 10% acrylamide gels by sodium dodecyl sulfate–polyacrylamide gel electrophoresis (SDS-PAGE) and transferred onto Hybond-ECL nitrocellulose membranes (Amersham Biosciences, Buckinghamshire, UK). Membranes were blocked with 5% skim milk in Tris-buffered saline containing 0.1% Tween-20 for 1 h at room temperature. The blots were probed using primary antibodies against FGFR1, FGFR2 (Santa Cruz Biotechnology, USA), FGFR3, FGFR4 (Abcam), phospho-FGFR1 (p-FGFR1), total-c-Met, phospho-c-Met (p-c-Met), total-AKT, phospho-AKT (p-AKT), total-ERK, phospho-ERK (p-ERK) (Cell Signaling Technology), or β-actin (Santa Cruz Biotechnology). Membranes were then incubated with horseradish peroxidase–conjugated anti-rabbit or anti-mouse secondary antibodies (GE Healthcare, Piscataway, NJ, USA). Bands were visualized using an enhanced chemiluminescence (ECL) kit (Amersham Biosciences, Buckinghamshire, UK) following the manufacturer’s protocols.

### 3-(4, 5-dimethylthiazol-2-yl)-2, 5-diphenyl tetrazolium bromide (MTT) assay

The MTT assay is based on conversion of MTT to insoluble MTT-formazan due to cleavage of the tetrazolium ring by the mitochondrial dehydrogenase enzymes of living cells. MTT solution (Amresco, Solon, USA) was added to each well. After 4 h of incubation, the medium was discarded, 100 µL of acidic isopropanol (0.1 N HCl in absolute isopropanol) was added, and the plate was gently shaken. Absorbance was measured using an enzyme-linked immunosorbent assay (ELISA) reader at a wavelength of 540 nm. Each sample was assayed in triplicate, and the experiment was repeated three times.

### Colony formation assay

Colony formation assay was performed in 6-well plates. A2780 and A2780-CP20 cells were plated at 1 × 10^2^/well, HeyA8 cells were 2 × 10^2^/well, and SKOV3ip1, SKOV3-TR, and HeyA8-MDR cells were 5 × 10^2^/well. EOC cells were treated with 0.1 µM and 1 µM AZD4547 for A2780 and for other cells, respectively. Cells were grown for 8 days to form visible colonies. The colonies were fixed with ice-cold methanol and stained with cell staining solution from a Cytoselect 24-well cell migration kit (Cell Biolabs). The plates were scanned on scanner (Samsung SL-J2160W) and the number of the colonies were counted. HeyA8 and HeyA8-MDR cells formed uncountable colonies and therefore the stained cells were extracted with extraction buffer from a Cytoselect 24-well cell migration kit (Cell Biolabs) and the optical density was measured at 560 nm.

### Migration assay

Migration assay was performed using a Cytoselect 24-well cell migration kit (Cell Biolabs) following the manufacturer’s protocols.

### Enzyme-linked immunosorbent assay (ELISA)

To analyze the secretion of the matrix metalloproteinase FGF19, the culture media were harvested and centrifuged to remove cellular debris. Media were concentrated by centrifugal filtration at 4,000 rpm for 20 min using an Amicon Ultra 10 K centrifugal filter tube (Millipore, Billerica, USA). ELISA kits were used to measure the concentration of human FGF19 (R&D Systems) as described by the manufacturer. Samples were measured in triplicate.

### Immunohistochemical analysis

Immunohistochemical studies were carried out on formalin-fixed, paraffin-embedded, 4-µm-thick tissue sections. Immunostaining for Ki-67 was performed with a BOND-MAX™ automated immunostainer (Leica Biosystems, Melbourne, Australia) and the BOND™ Polymer refined detection kit (Vision Biosystems, Melbourne, Australia). Briefly, antigen retrieval was performed at 97 °C for 20 min in ER1 buffer. After blocking endogenous peroxidase activity with 3% hydrogen peroxidase for 10 min, primary antibody incubation was performed for 15 min at room temperature using an antibody dilution of 1:200. Apoptotic cells were analyzed by TUNEL assay using the DeadEnd colormetric TUNEL assay kit (Promega, Madison, WI) as previously described.

### Animal care and development of in vivo models including established cell line and PDX

Female BALB/c nude mice were purchased from OrientBio, Seongnam, Korea. This study was reviewed and approved by the Institutional Animal Care and Use Committee (IACUC) of the Samsung Biomedical Research Institute (protocol No. H-A9-003), which is an Association for the Assessment and Accreditation of Laboratory Animal Care International (AAALAC International)–accredited facility that abides by the Institute of Laboratory Animal Resources (ILAR) guide (IRB No. 2010-04-004).

To establish intraperitoneal xenograft models, A2780 (1 × 10^6^ cells/0.2 mL HBSS), SKOV3ip1 (1 × 10^6^ cells/0.2 mL HBSS), A2780-CP20 (1 × 10^6^ cells/0.2 mL HBSS), and SKOV3-TR (1 × 10^6^ cells/0.2 mL HBSS) were injected into the peritoneal cavity of mice. For the PDX model of EOC, patient tumor specimens retrieved from the operation room were sliced into small pieces (under 2–3 mm), implanted into the sub-renal capsule of the left mouse kidney, and propagated by serial transplantation [14]. Tumor fragments were implanted in a minimum of five mice, depending on tumor tissue availability.

Mice used in experiments were 6–8 weeks old. Mice (*n* = 10 per group) were monitored daily for tumor development and sacrificed on day 30 ~ 35 after injection of cancer cells, on day 60 ~ 70 (PDX model), or if mice seemed moribund. We recorded the total body weight, tumor weight, and the number of tumor nodules. Tumors were fixed in formalin and embedded in paraffin or snap-frozen in OCT compound (Sakura Finetek Japan, Tokyo, Japan) in liquid nitrogen.

### Signaling pathway analysis

We previously performed a pharmacogenomics analysis of patient-derived tumor cells in gynecologic cancers [15]. To determine the pathway signature activity between AZD4547-resistant and -sensitive patient-derived cells, we used the Gene Set Enrichment Analysis (GSEA) algorithm. GSEA is a computational method that evaluates and compares the activities of predefined gene sets based on a gene expression matrix between two groups. We performed GSEA using the following parameters: *Gene sets database*: “c2.all.v2022.1.Hs.symbols.gmt,” *Number of permutations*: “1000,” and *Permutation type*: “gene_set.”

### Data analysis

The Mann–Whitney U test was used to compare differences among the groups and to evaluate significance for both in vitro and in vivo assays. All statistical tests performed were two-sided, and *p*-values less than 0.05 were considered statistically significant. Prism (GraphPad, CA, USA) was used for all statistical analyses.

## Results

### Effects of AZD4547, a selective FGFR 1–3 inhibitor, on cell viability of EOC cells

**Fig. 1:**
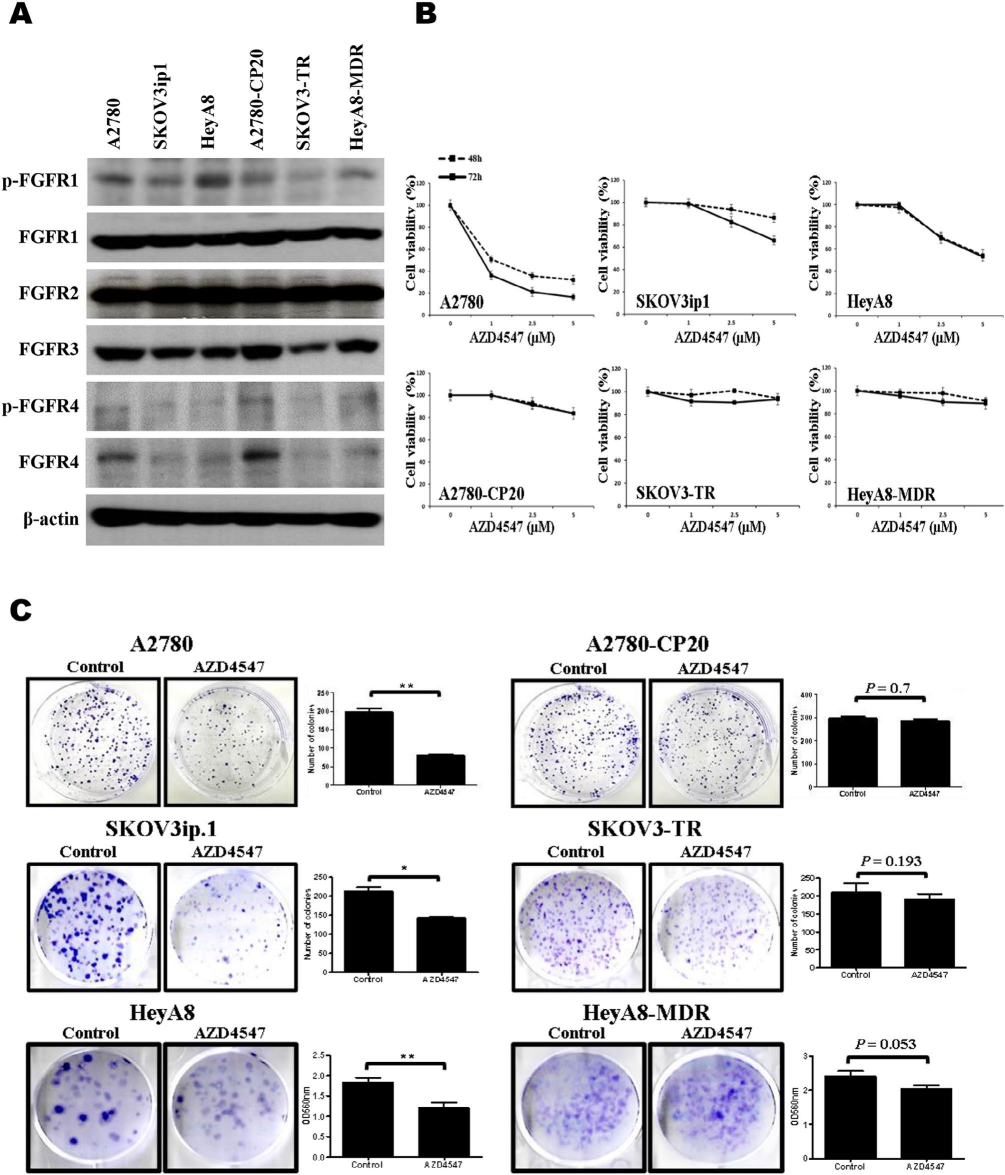
Cytotoxic effects of AZD4547 in epithelial ovarian cancer (EOC) cell lines. (**A**) Expression levels of FGFRs in EOC cell lines were analyzed by western blot. (**B**) EOC cells were treated with AZD4547 for the indicated times, and cell viability was evaluated by MTT assay. (**C**) Colony formation assay was performed with EOC cell lines. AZD4547 was incubated for 8 days until colony was formed. Colony was stained and counted or stained colonies were measured by the optical density at 560 nm after extraction of colonies as described in “Methods”

A2780-CP20 cells are cisplatin-resistant cells derived from A2780 cells, and HeyA8-MDR and SKOV3-TR cells are paclitaxel-resistant cells obtained from HeyA8 and SKOV3 cells, respectively. High expression of FGFR1–3 was observed in EOC cells, as shown in Fig. 1A. There were no significant differences in FGFR1–3 expression level between cells; however, FGFR4 was strongly expressed only in A2780 and A2780-CP20 cells.

In chemo-sensitive cells, such as A2780, HeyA8, and SKOV3ip1 cells, AZD4547 significantly reduced cell proliferation at 48 and 72 h in a dose-dependent manner (Fig. 1B). However, AZD4547 had no effect in chemo-resistant A2780-CP20, HeyA8-MDR, and SKOV3-TR cells at any time point. We performed colony formation assay with the EOC cells. AZD4547 reduced colony formation by 60% for A2780 cells and 33 ~ 35% for SKOV3ip1 and HeyA8 cells (Fig. 1C). In contrast with the chemo-sensitive cells, AZD4547 had no effect in chemo-resistant A2780-CP20, HeyA8-MDR, and SKOV3-TR cells.

### Effects of AZD4547 on EOC cell migration

**Fig. 2:**
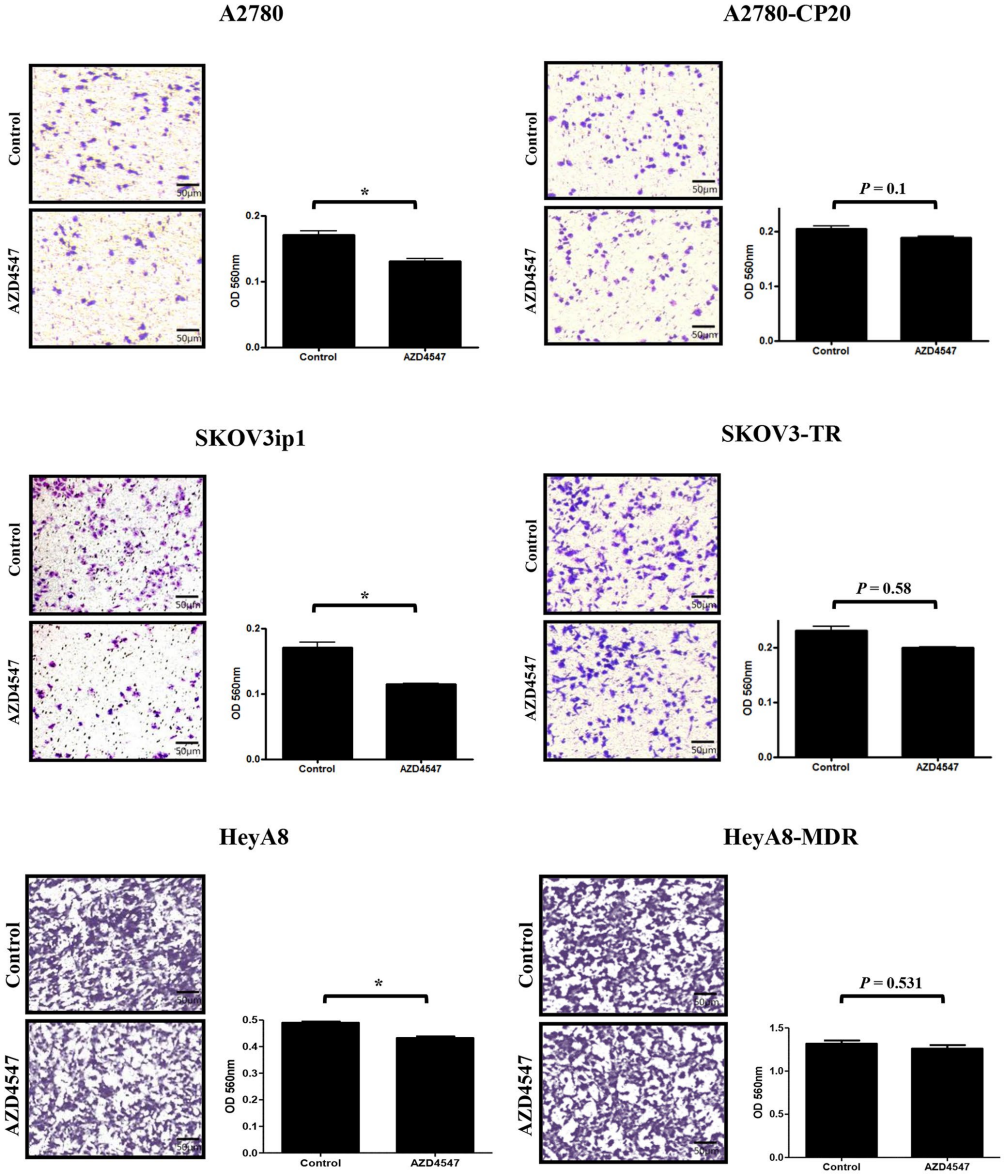
The effects of AZD4547 on cell migration of EOC cell lines after 24 h. Migration assay was performed using the Cytoselect 24-well cell migration kit after AZD4547 treatment in EOC cell lines (A2780: 0.1 µM, SKOV3ip1, HeyA8, A2780-CP20, SKOV3-TR, and HeyA8-MDR: 1 µM) (*p < 0.05)

To assess the effect of AZD4547 on cell migration, non-lethal doses of AZD4547 were administered to A2780, A2780-CP20, SKOV3ip1, SKOV3-TR, HeyA8, and HeyA8-MDR cells (Fig. 2). AZD4547 significantly inhibited the migration of A2780 (*p* = 0.038) and SKOV3ip1 (*p* = 0.03) cells and slightly inhibited HeyA8 cells (*p* = 0.028). These effects were not observed in A2780-CP20 (*p* = 0.1), SKOV-TR (*p* = 0.58), and HeyA8-MDR (*p* = 0.531) cells, similar to the results observed in MTT assay.

### Effect of AZD4547 on tumor growth in EOC cell line intraperitoneal xenografts and PDX mouse models

**Fig. 3:**
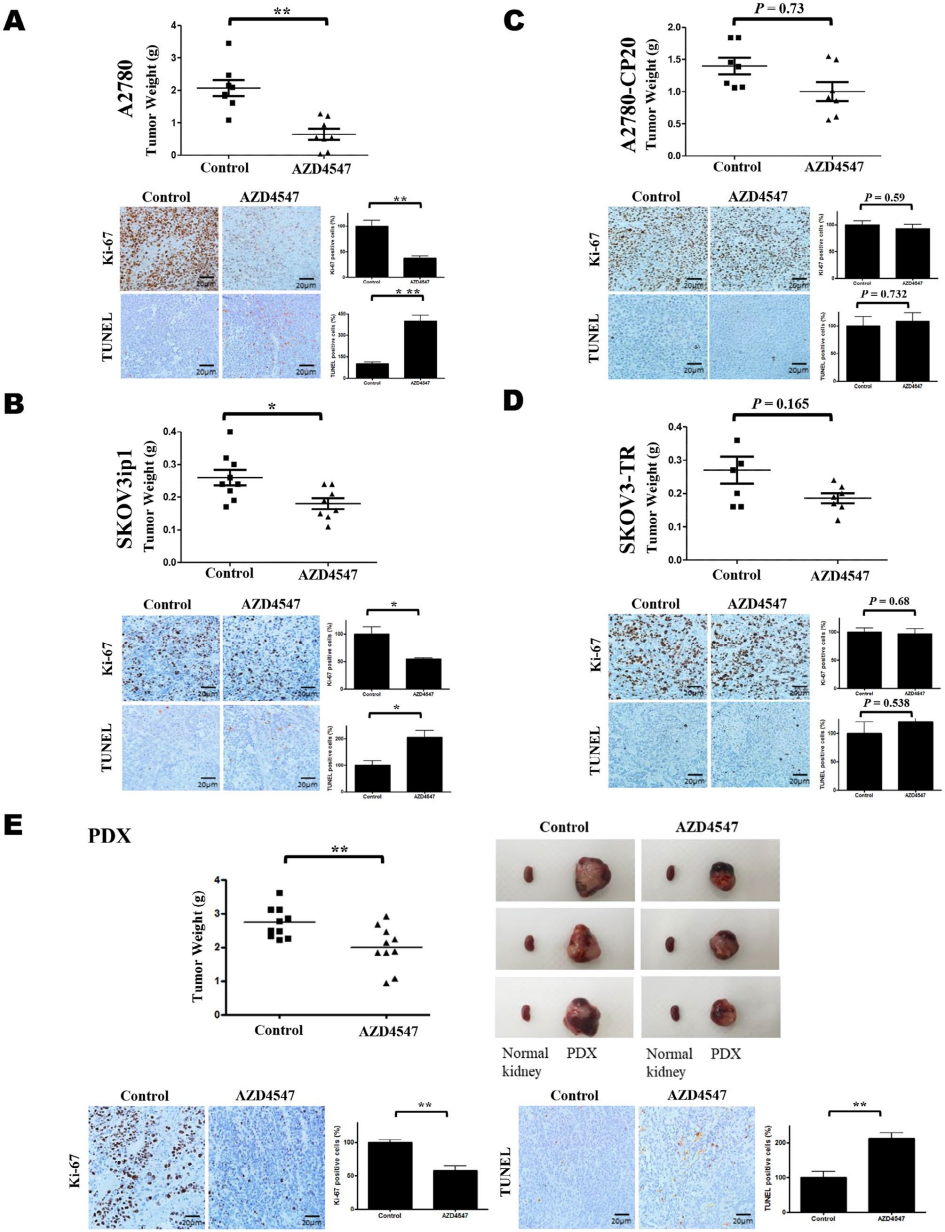
AZD4547 inhibited tumor growth in ovarian cancer- and EOC patient–derived xenograft models. (**A**) A2780-, (**B**) SKOV3ip1-, (**C**) A2780-CP20-, and (**D**) SKOV3-TR-derived xenograft and (**E**) EOC PDX mice. Groups treated with AZD4547 (*n* = 10) exhibited significantly decreased tumor weight compared with the control group (*n* = 10). Top panels show tumor weights and bottom panels show tumor cell proliferation based on Ki-67 immunohistochemistry of harvested tumor tissues (*n* = 5 for each group) and TUNEL assays (*p < 0.05). In (**E**), images of tumors obtained from PDX mice are compared with kidney tissue obtained from the same mouse

To investigate the clinical relevance of the in vitro results, we conducted in vivo experiments with EOC intraperitoneal xenograft mouse models. A2780, A2780-CP20, SKOV3ip1, and SKOV3-TR EOC cells were implanted into peritoneal cavities of female nude mice, and treatment with AZD4547 (25 mg/kg daily p.o.) was started at day 7 after injection of the cells [16]. In the A2780 and SKOV3ip1 models, the tumor weight of the AZD4547-treated group was significantly decreased compared with the controls (Fig. 3A and B, *p* = 0.01, and *p* = 0.015, respectively). Treatment of AZD4547 had no effects in SKOV3-TR- and A2780-CP20-derived xenograft animal models (Fig. 3C and D *p* = 0.165, *p* = 0.73, respectively).

To validate the results of the in vitro studies, we evaluated the effects of AZD4547 on cell proliferation and apoptosis in tumor tissues obtained from xenograft mice by immunohistochemical staining for Ki-67 and TUNEL assay, respectively. The number of Ki-67-positive cells in the A2780 and SKOV3ip1 models was significantly lower in tumors from mice treated with AZD4547 than in tumors from controls (Fig. 3A and B, respectively). These effects were not observed in A2780-CP20 and SKOV3-TR mouse models (Fig. 3C and D, *p* = 0.59 and *p* = 0.68, respectively). TUNEL assay results showed that the number of apoptotic cells was significantly higher in AZD4547-treated tumors from A2780 and SKOV3ip1 mouse models but not in the A2780-CP20 and SKOV3-TR mouse models (Fig. 3, *p* = 0.732 and *p* = 0.538, respectively).

We also examined the effects of AZD4547 in PDX models of EOC. Our group previously developed PDX models of EOC [14]. We selected a patient diagnosed with FIGO stage IIIC ovarian cancer (high-grade serous carcinoma); the PDX model was established using the patient’s tumor samples (OV-71) obtained during maximal cytoreductive surgery. The patient was a 58-year-old female who underwent 3 cycles of neoadjuvant chemotherapy with tri-weekly intravenous carboplatin and paclitaxel treatments, followed by an interval debulking surgery in 2015, an optimal debulking that resulted in residual disease of less than 1 cm. Afterward, adjuvant tri-weekly intravenous carboplatin and paclitaxel treatments were administered for 5 cycles. There was no evidence of disease up to December 2019 (disease-free survival for 4 years 8 months). Treatment with AZD4547 significantly decreased tumor weights in the PDX mouse model (*p* = 0.009). The numbers of Ki-67- and TUNEL-positive cells were reduced and increased, respectively, indicating inhibition of cell proliferation and increase of apoptosis in AZD4547-treated tumors (Fig. 3E).

### Expression of downstream molecules of the FGF/FGFR pathway after AZD4547 treatment for EOC and PDX mice

**Fig. 4:**
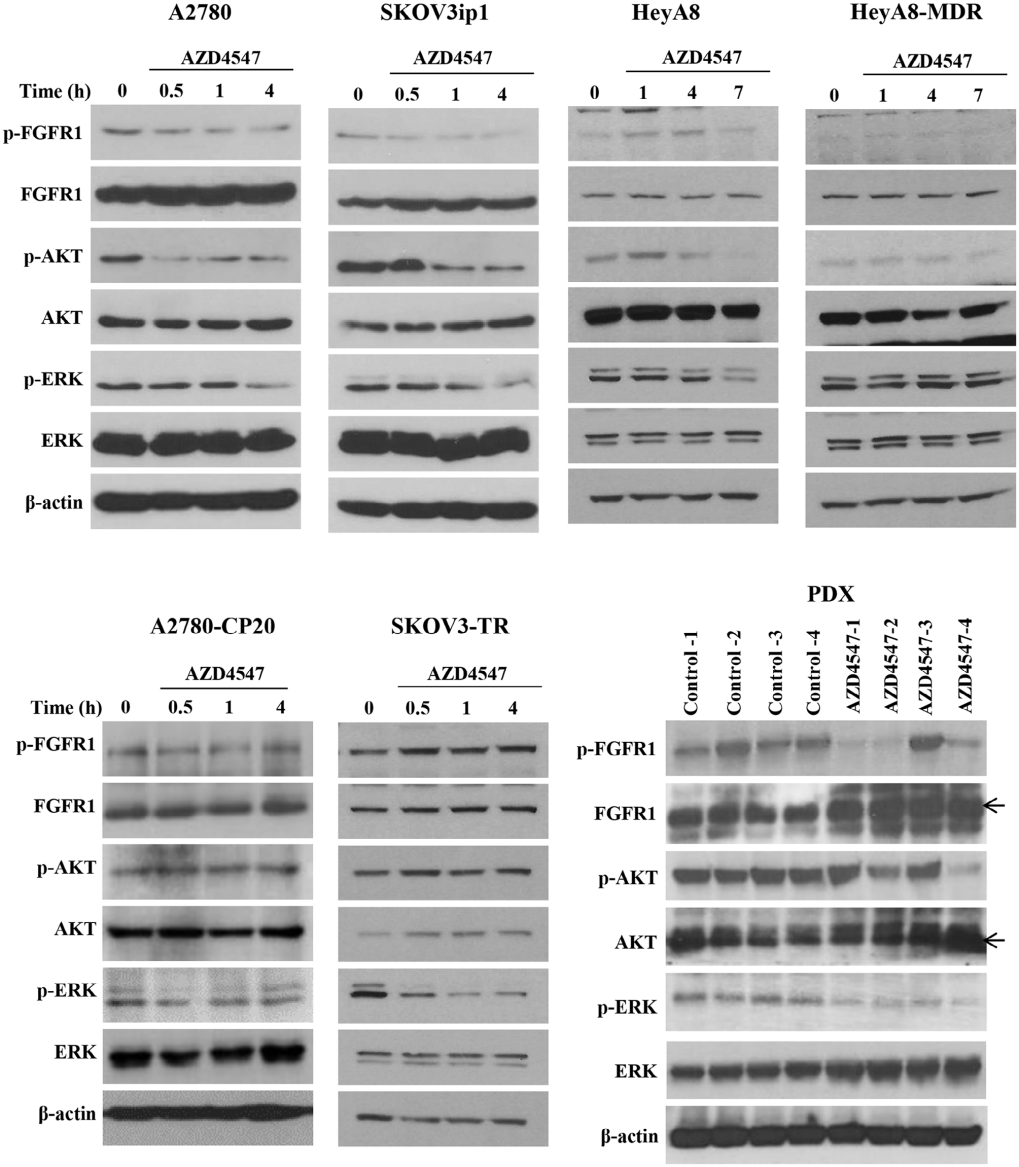
Inhibition of FGFR1 activation and downstream signaling by AZD4547 were dependent on the EOC cell lines and chemo-sensitive PDX. AZD4547 was applied for different durations in EOC cells. Phospho-FGFR1 and phosphorylation of downstream signaling proteins including p-AKT and p-ERK were investigated in cell lysates and tumor lysates obtained from AZD4547-treated PDX mice compared with control-treated mice by western blot analysis. β-actin was used as a loading control

To evaluate the downstream signaling pathway involved in the anti-cancer activity of AZD4547 in EOC cells, we assessed the FGF/FGFR pathway by western blot analysis. AZD4547 (1 µM) was applied for different times as indicated in the figure. Expression levels of phosphorylated FGFR1/FGFR1 and the downstream molecules including p-AKT/AKT and p-ERK/ERK [7] were significantly decreased 4 and 7 h after AZD4547 treatment in A2780 and SKOV3ip1 cells and HeyA8 cells, respectively (Fig. 4). The downstream molecules were also investigated in chemo-resistant cells (Fig. 4). Treatment of AZD4547 had no effect on the downstream signaling molecules in the chemo-resistant cells. Except, p-ERK was reduced in SKOV3-TR cells. We also determined the FGF/FGFR pathway in tumor lysates obtained from AZD4547-treated PDX mice and control mice using western blot analysis. Expression levels of phosphorylated FGFR1, p-AKT/AKT, and p-ERK/ERK were inhibited by AZD4547.

### Combination effects of AZD4547 and c-Met inhibitor on EOC cells expressing high levels of c-Met

**Fig. 5:**
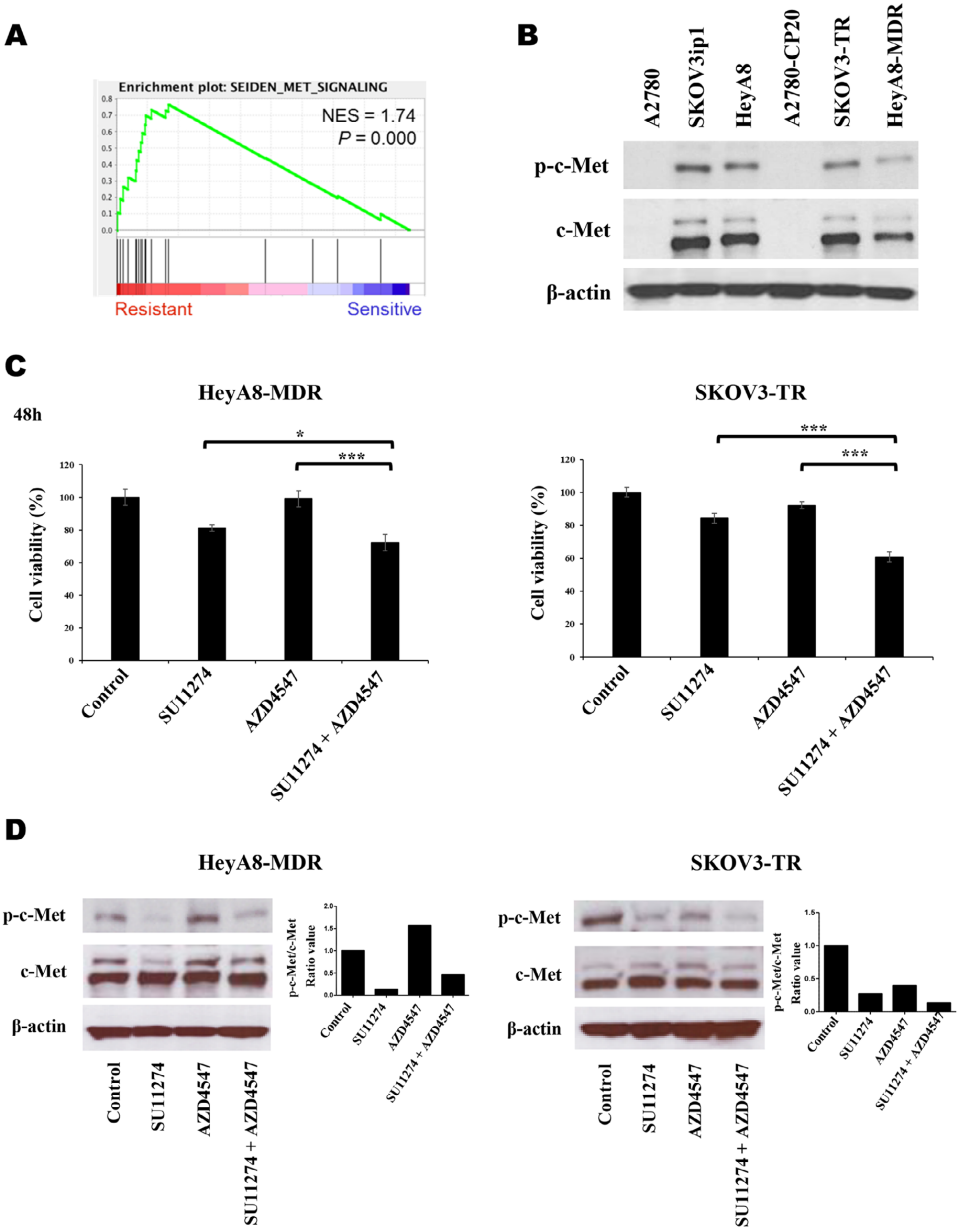
Combination of c-Met inhibitor with AZD4547 induced cell death of AZD4547-resistant cells overexpressing c-Met. (**A**) To determine the pathway signature activity, Gene Set Enrichment Analysis (GSEA) was performed in AZD4547-resistant and -sensitive patient-derived cells. (**B**) Expression of p-c-Met and c-Met was analyzed by western blotting in EOC cells. (**C**) Cell viability was measured in cells treated with SU11274, a c-Met-specific inhibitor, AZD4547, and the combination of SU11274 with AZD4547 in HayA8-MDR and SKOV3-TR cells for 48 h (*p < 0.05, **p < 0.001). (**D**) Expression of p-c-Met and c-Met was analyzed by western blot in lysates from the same experimental conditions as in (**C**). Expression of p-c-Met was normalized by total c-Met expression using Image J, and the result is shown in the right panel

We next explored a strategy to overcome resistance against AZD4547 in chemo-resistant EOC cells. Using GSEA, we found that genes involved in the c-Met-related signaling pathway were highly expressed in AZD4547-resistant patient-derived cells compared with AZD4547-sensitive cells from our previous study (Fig. 5A, [15]). The expression of c-Met was examined in EOC cells by western blot analysis. As shown in Fig. 5B, c-Met was highly expressed in AZD4547-resistant HeyA8-MDR and SKOV3-TR cells and in AZD4547-sensitive HeyA8 and SKOV3ip1 cells. However, c-Met was not detected in A2780 and A2780-CP20 cells by western blotting. The combination of AZD4547 and a c-Met inhibitor, SU11274, was assessed for its ability to overcome AZD4547 resistance. SU11274 was applied either alone or with AZD4547 in HeyA8-MDR and SKOV3-TR cells, and cell viability was measured (Fig. 5C). AZD4547 at 5 µM had no effect on cell death in HeyA8-MDR and SKOV3-TR cells. However, combination with SU11274 led to cell death at ~ 28% and ~ 40% in HeyA8-MDR and SKOV3-TR cells, respectively, compared with the control treatment. SU11274 alone resulted in ~ 20% of cell death in both cell lines. Kim et al. [17] reported that treatment with AZD4547 led to overexpression of c-Met, resulting in acquired resistance to AZD4547. Therefore, the expression of p-c-Met and total c-Met was assessed (Fig. 5D). Treatment with AZD4547 alone induced p-c-Met expression in HeyA8-MDR cells. However, SU11274 alone and in combination with AZD4547 inhibited phosphorylation of c-Met in HeyA8-MDR cells (Fig. 5D). In SKOV3-TR cells, single treatment with AZD4547 or SU11274 inhibited the phosphorylation of c-Met, and the combination of these inhibitors further inhibited p-c-Met (Fig. 5D). Taken together, these results indicate that inhibition of p-c-Met in combination with AZD4547 induced cell death in AZD4547-resistant cells.

### Enhanced anti-tumor effects by blocking FGF19 or FGFR4 in AZD4547-resistant A2780-CP20

**Fig. 6:**
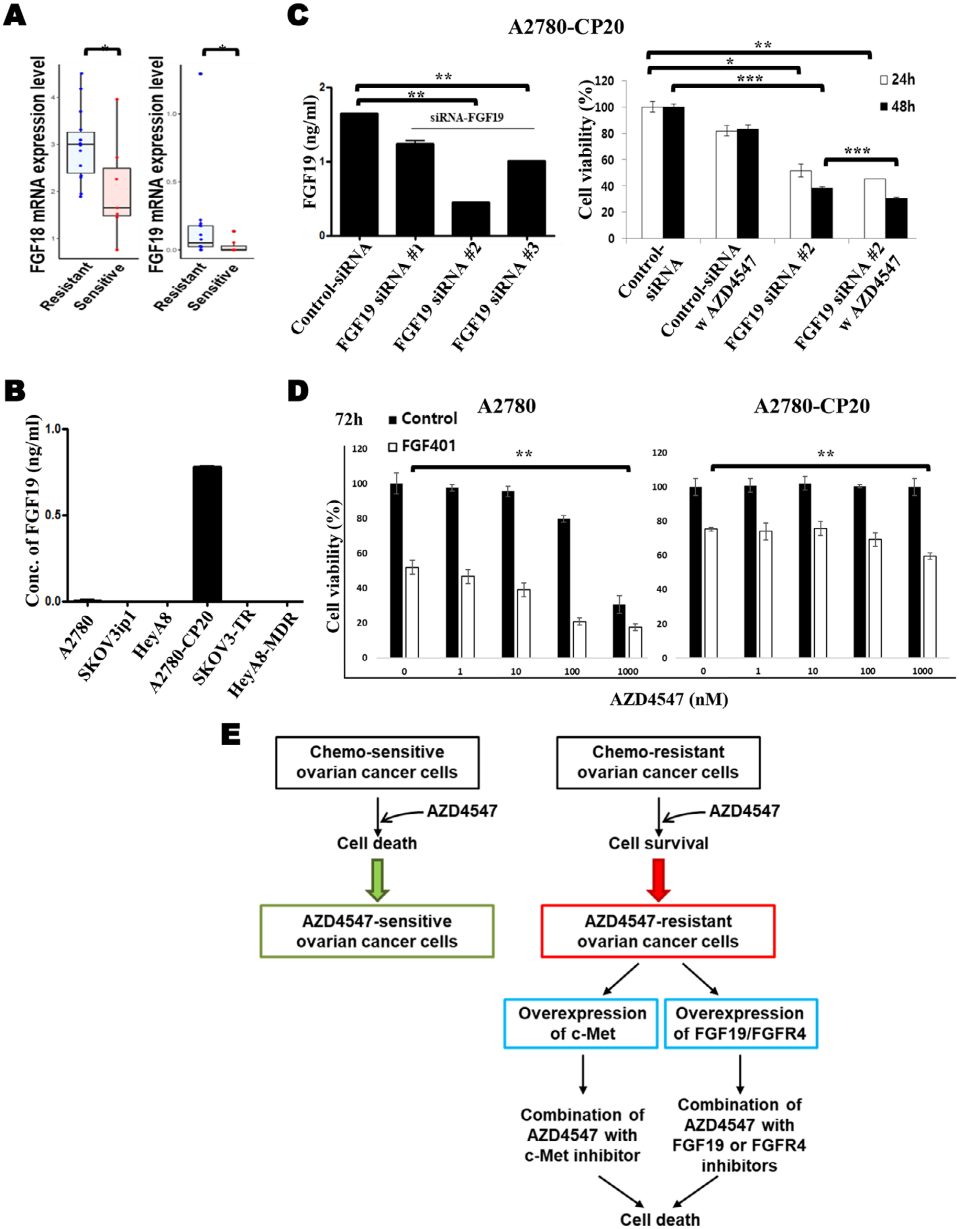
Downregulation of FGF19 reduced cell viability in A2780-CP20 cells. (**A**) Pharmacogenomic analysis of FGF18 and FGF19 was performed in the AZD4547-resistant group compared with the AZD4547-sensitive group (*p < 0.05). (**B**) The level of secreted FGF19 in EOC cell lines was assessed by ELISA. (**C**) A2780-CP20 cells were transfected with FGF19-siRNA (50 nM) or control siRNA and incubated for 72 h. Transfected cells were then exposed to 1 µM AZD4547 for 24 or 48 h, and cell viability was assessed by MTT assay (*p < 0.05, **p < 0.001). (**D**) Combination effect of AZD4547 with FGF401, an FGFR4 inhibitor, was assessed in A2780 and A2780-CP20 cells by MTT assay at 48 h. (**E**) A schematic representation of targeted therapy of FGF/FGFR in chemo-sensitive and chemo-resistant EOCs

We previously performed a pharmacogenomics analysis of patient-derived tumor cells in gynecologic cancers and demonstrated the potential utility of rapid drug screening combined with genomic profiling for precision treatment of gynecologic cancers [15]. Using these data, we analyzed responses to AZD4547 based on the mRNA expression of FGFs (FGF1–23) and FGFRs (FGFR1–4) in EOC patient tumor samples to test for the possible role of these genes as predictive biomarkers for AZD4547. As shown in Fig. 6A, the mRNA expression levels of FGF18 and FGF19 were significantly higher in AZD4547-resistant samples. No significant differences were found in the expression levels of the other FGFs. Because FGF19 is the FGF that signals through FGFR4 in humans [18] and because the FGF19/FGFR4 axis has a critical role in some solid tumors [19], we focused on FGF19 and FGFR4 in EOC. We measured FGF19 expression level in the conditioned media from EOC cells using ELISA and found that it was exclusively elevated in A2780-CP20 cells (Fig. 6B), which showed resistance to AZD4547 in vitro and in vivo as described above.

To study the effect of FGF19 on the cell viability of EOC, FGF19 was knocked down with siRNA. On transfection with three FGF19-specific siRNAs (#1, #2, and #3), the expression level of FGF19 was significantly reduced in the conditioned media (Fig. 6C). The viability of A2780-CP20 cells significantly decreased after treatment with one of the FGF19 siRNAs (#2). Notably, 48 h of treatment with AZD4547 on FGF19 siRNA (#2)–transfected cells resulted in significantly decreased cell viability (Fig. 6C). We investigated the effect of a combination of AZD4547 (a selective FGFR1–3 inhibitor) and FGF401 (a selective FGFR4 inhibitor, roblitinib) on cell proliferation of A2780 and A2780-CP20 cells (Fig. 6D). Single treatment with FGF401 induced cell death in both cell lines. While AZD4547 alone had anti-cancer activity, its combination with FGF401 further inhibited cell viability in A2780 cells. A high dose of AZD4547 had no effect on cell viability of A2780-CP20 but combination with FGF401 induced cell death, suggesting that the combination of AZD4547 with FGF401 enhances cell death in FGF19/FGFR4-overexpressing cell lines.

## Discussion

In this study, we found that inhibition of FGFR by AZD4547, a selective FGFR1–3 inhibitor, showed potential therapeutic effects on EOC cells but not chemo-resistant cells such as A2780-CP20, HeyA8-MDR, and SKOV3-TR cells. Analysis of signaling pathways by GSEA and gene expression analysis revealed that AZD4547-resistant tumor cells had enhanced c-Met signaling and higher FGF18 and FGF19 mRNA expression compared with AZD4547-sensitive cells. Inhibition of c-Met, FGF19, or FGFR4 by inhibitors or siRNA sensitized AZD4547-resistant EOC cells to AZD4547. These findings suggest that activated c-Met-related signals and high expression of FGF19 and FGFR4 may be biomarkers in predicting responses to AZD4547, and targeting these molecules may be a promising strategy to enhance the effects of AZD4547 in EOCs, particularly in AZD4547-resistant EOCs.

c-Met is a receptor tyrosine kinase (RTK) and an oncogenic protein that facilitates cell proliferation and invasion [20]. Activation of c-Met by binding of HGF, its ligand, induces cell proliferation and migration. c-MET is highly expressed in various types of cancer including ovarian, breast, colon, and prostate cancers and melanomas. Previous studies showed that enhanced expression of c-Met was related with drug resistance [20]. The therapeutic effect of AZD4547 is dependent on expression of c-Met. Kim et al. found that c-Met amplification led to acquired resistance to AZD4547 in lung cancer cells [17]. Cells expressing high FGFR1 and FGFR2 levels were resistant to c-Met inhibitor, and knockdown of FGFR1/2 led cells to be sensitive to c-Met inhibitor. These findings suggest that there are certain correlations between c-Met and FGFR expression in resistance to their inhibitors. Therefore, we measured p-c-Met expression in AZD4547-treated HeyA8-MDR and SKOV3-TR cells. Single treatment with AZD4547 induced p-c-Met in HeyA8-MDR cells but not in SKOV3-TR cells. AZD4547 alone inhibited p-c-Met in SKOV3-TR cells. Co-treatment with AZD4547 and SU11274 further inhibited p-c-Met in these cells and resulted in reduced cell viability. Taken together, these results show that, while inhibition of c-Met facilitates cell death of AZD4547-resistant c-Met-overexpressing EOC cells, the mechanism might be cell-type specific.

Several studies have investigated clinical features based on genetic aberrations or differing expression levels of FGFs or FGFRs in EOCs. For example, *FGF3* amplification (copy numbers), which is observed in 20% of EOC samples [21], was significantly associated with FIGO stage but not with survival [10]. Another study found significant differences between FGF1 mRNA level and overall survival in 42 patients with advanced stage high-grade serous ovarian carcinoma, suggesting that *FGF1* amplification may be a poor prognostic factor [9]. Although FGFR amplification or overexpression was not associated with clinical characteristics in patients with EOC as opposed to FGFs, inhibition of FGFRs exhibited therapeutic effects on EOC cells. For example, a study demonstrated that FGFR1/2 inhibition increased cisplatin sensitivity in EOC cell lines [6]. Those authors also found that decreased expression of FGFR2/3/4 with shRNA significantly inhibited cell proliferation by 40–80% compared with the controls. Na et al. reported that AZD4547 inhibited tumor growth by reducing cell viability, migration, invasion, and angiogenesis in EOCs and by attenuating self-renewal in cancer stem cells [13]. We also observed anti-tumor effects of AZD4547 on EOC cells (A2780 and SKOV3ip1)* in vitro* (cell viability and migration) and in vivo (both in cell line xenografts and a PDX model). These findings suggest the use of AZD4547 in the treatment of drug-sensitive EOCs [9, 11].

Cole et al. suggested that broad-spectrum FGFR inhibitors do not have the desired therapeutic effects on EOC cells, since reduction of FGFR1 expression by shRNA increased SKOV3 cell number [6]. In this study, we also found that the anti-tumor effects of AZD4547 were different in EOC cells. Therefore, we investigated the AZD4547-resistance mechanism based on the differential expression of FGF/FGFRs. Expression of FGF18 and FGF19 was enhanced in AZD4547-resistant EOC cells compared with AZD4547-sensitive cells. Among all FGFs, FGF3 and FGF19 are nearly exclusively expressed in EOCs but are rarely or not expressed in normal tissues [6]. FGF18 was also highly expressed in malignant tissue as well as normal ovarian tissue, suggesting that FGF19 may be more ovarian cancer–specific compared with FGF18. For this reason, we considered FGF19 as an important biomarker to predict the response to AZD4547. Since FGFR4 is the receptor for FGF19, we studied both FGF19 and FGFR4. To investigate the role of activated FGF19 or FGFR4 in A2780-CP20 cells with regard to AZD4547 resistance, FGF19 siRNA or FGFR4 inhibitor was used to block these signaling pathways. We found that A2780-CP20 cells became sensitive to AZD4547 when co-treated with either FGF19-specific siRNA or FGFR4 inhibitor. These findings suggest that FGF19 or FGFR4 may be a promising therapeutic target in A2780-CP20 cells. Several studies have examined resistance mechanisms to AZD4547, suggesting FGFR1 mutations or FGFR isoforms as possible explanations for AZD4547 resistance [22–24]. The role of the FGF/FGFR pathway has been studied in several solid tumors, and diverse findings have been observed across tumor types. For example, serum FGF21 level was associated with disease recurrence in urothelial cancer [25]. FGF2 was associated with the growth of lung adenocarcinoma [26] and pancreatic ductal adenocarcinoma [27]. In hepatocellular carcinoma, the FGF19/FGFR4 axis was reported to be an oncogenic driver pathway, suggesting it as a promising target [19, 28]. Moreover, the FGF19/FGFR4 axis was correlated with poor prognosis in advanced high-grade serous ovarian cancer [29, 30]. These findings should be considered when designing future clinical trials with various FGF/FGFR inhibitors in different types of tumors [7, 31, 32].

## Conclusions

FGFR1-3 inhibition with AZD4547 showed significant anti-tumor effects in EOC models but not in cells highly expressing c-Met and FGF19/FGFR4, which can be treated with either c-Met and/or FGF19/FGFR4 inhibition in combination with AZD4547. We suggest that c-Met and FGF19/FGFR4 levels can serve not only as a potential predictive biomarker for AZD4547 treatment response, but also as therapeutic targets for resistant EOCs.

### Electronic supplementary material

Below is the link to the electronic supplementary material.

Supplementary Material 1

## Data Availability

Not applicable.

## Abbreviations

EOC: Epithelial ovarian cancer
FGFR: Fibroblast growth factor receptor
PDX: Patient-derived xenograft
RTK: Receptor tyrosine kinase
MTT: 3-(4, 5-Dimethylthiazol-2-yl)-2, 5-diphenyl tetrazolium bromide
ELISA: Enzyme-Linked Immunosorbent Assay
siRNA: Small Interfering RNA

## References

[1] Reid BM, Permuth JB, Sellers TA (2017). Epidemiology of ovarian cancer: a review. Cancer Biol Med.

[2] Momenimovahed Z, Tiznobaik A, Taheri S, Salehiniya H (2019). Ovarian cancer in the world: epidemiology and risk factors. Int J Womens Health.

[3] Markham MJ, Wachter K, Agarwal N, Bertagnolli MM, Chang SM, Dale W, Diefenbach CSM, Rodriguez-Galindo C, George DJ, Gilligan TD, et al. Clinical Cancer advances 2020: Annual Report on Progress Against Cancer from the American Society of Clinical Oncology. J Clin Oncol. 2020;38(10):1081. 10.1200/JCO.19.03141 32013670

[4] Moschetta M, George A, Kaye SB, Banerjee S (2016). BRCA somatic mutations and epigenetic BRCA modifications in serous ovarian cancer. Ann Oncol.

[5] Johnson DE, Williams LT (1993). Structural and functional diversity in the FGF receptor multigene family. Adv Cancer Res.

[6] Cole C, Lau S, Backen A, Clamp A, Rushton G, Dive C, Hodgkinson C, McVey R, Kitchener H, Jayson GC (2010). Inhibition of FGFR2 and FGFR1 increases cisplatin sensitivity in ovarian cancer. Cancer Biol Ther.

[7] Chae YK, Ranganath K, Hammerman PS, Vaklavas C, Mohindra N, Kalyan A, Matsangou M, Costa R, Carneiro B, Villaflor VM (2017). Inhibition of the fibroblast growth factor receptor (FGFR) pathway: the current landscape and barriers to clinical application. Oncotarget.

[8] Helsten T, Elkin S, Arthur E, Tomson BN, Carter J, Kurzrock R (2016). The FGFR Landscape in Cancer: analysis of 4,853 tumors by Next-Generation sequencing. Clin cancer Research: Official J Am Association Cancer Res.

[9] Birrer MJ, Johnson ME, Hao K, Wong KK, Park DC, Bell A, Welch WR, Berkowitz RS, Mok SC (2007). Whole genome oligonucleotide-based array comparative genomic hybridization analysis identified fibroblast growth factor 1 as a prognostic marker for advanced-stage serous ovarian adenocarcinomas. J Clin Oncol.

[10] Rosen A, Sevelda P, Klein M, Dobianer K, Hruza C, Czerwenka K, Hanak H, Vavra N, Salzer H, Leodolter S (1993). First experience with FGF-3 (INT-2) amplification in women with epithelial ovarian cancer. Br J Cancer.

[11] Sun Y, Fan X, Zhang Q, Shi X, Xu G, Zou C (2017). Cancer-associated fibroblasts secrete FGF-1 to promote ovarian proliferation, migration, and invasion through the activation of FGF-1/FGFR4 signaling. Tumour Biol.

[12] Zheng J, Zhang W, Li L, He Y, Wei Y, Dang Y, Nie S, Guo Z (2022). Signaling pathway and small-molecule drug Discovery of FGFR: a Comprehensive Review. Front Chem.

[13] Na YR, Kim JY, Song CH, Kim M, Do YT, Vo TTL, Choi E, Ha E, Seo JH, Shin SJ. The FGFR Family Inhibitor AZD4547 Exerts an Antitumor Effect in Ovarian Cancer Cells. Int J Mol Sci. 2021;22(19). 10.3390/ijms221910817 PMC8509426 34639155

[14] Heo EJ, Cho YJ, Cho WC, Hong JE, Jeon HK, Oh DY, Choi YL, Song SY, Choi JJ, Bae DS (2017). Patient-derived xenograft models of epithelial ovarian Cancer for preclinical studies. Cancer Res Treat.

[15] Sa JK, Hwang JR, Cho YJ, Ryu JY, Choi JJ, Jeong SY, Kim J, Kim MS, Paik ES, Lee YY (2019). Pharmacogenomic analysis of patient-derived tumor cells in gynecologic cancers. Genome Biol.

[16] Zhang J, Zhang L, Su X, Li M, Xie L, Malchers F, Fan S, Yin X, Xu Y, Liu K (2012). Translating the therapeutic potential of AZD4547 in FGFR1-amplified non-small cell lung cancer through the use of patient-derived tumor xenograft models. Clin cancer Research: Official J Am Association Cancer Res.

[17] Kim SM, Kim H, Yun MR, Kang HN, Pyo KH, Park HJ, Lee JM, Choi HM, Ellinghaus P, Ocker M (2016). Activation of the Met kinase confers acquired drug resistance in FGFR-targeted lung cancer therapy. Oncogenesis.

[18] Heinzle C, Erdem Z, Paur J, Grasl-Kraupp B, Holzmann K, Grusch M, Berger W, Marian B (2014). Is fibroblast growth factor receptor 4 a suitable target of cancer therapy?. Curr Pharm Des.

[19] Subbiah V, Pal SK (2019). Precision Oncology for Hepatocellular Cancer: slivering the liver by FGF19-FGF4-KLB pathway inhibition. Cancer Discov.

[20] Kim HJ. Therapeutic strategies for ovarian Cancer in point of HGF/c-MET targeting. Med (Kaunas). 2022;58(5). 10.3390/medicina58050649 PMC9147666 35630066

[21] Hruza C, Dobianer K, Beck A, Czerwenka K, Hanak H, Klein M, Leodolter S, Medl M, Mullauer-Ertl S, Preiser J (1993). HER-2 and INT-2 amplification estimated by quantitative PCR in paraffin-embedded ovarian cancer tissue samples. Eur J Cancer.

[22] Fu W, Chen L, Wang Z, Kang Y, Wu C, Xia Q, Liu Z, Zhou J, Liang G, Cai Y (2017). Theoretical studies on FGFR isoform selectivity of FGFR1/FGFR4 inhibitors by molecular dynamics simulations and free energy calculations. Phys Chem Chem Phys.

[23] Liang D, Chen Q, Guo Y, Zhang T, Guo W (2017). Insight into resistance mechanisms of AZD4547 and E3810 to FGFR1 gatekeeper mutation via theoretical study. Drug Des Devel Ther.

[24] Ryan MR, Sohl CD, Luo B, Anderson KS (2019). The FGFR1 V561M gatekeeper mutation drives AZD4547 resistance through STAT3 activation and EMT. Mol Cancer Res.

[25] Bahleda R, Italiano A, Hierro C, Mita A, Cervantes A, Chan N, Awad M, Calvo E, Moreno V, Govindan R (2019). Multicenter Phase I study of Erdafitinib (JNJ-42756493), oral pan-fibroblast growth factor receptor inhibitor, in patients with Advanced or Refractory Solid tumors. Clin cancer Research: Official J Am Association Cancer Res.

[26] Hegab AE, Ozaki M, Kameyama N, Gao J, Kagawa S, Yasuda H, Soejima K, Yin Y, Guzy RD, Nakamura Y (2019). Effect of FGF/FGFR pathway blocking on lung adenocarcinoma and its cancer-associated fibroblasts. J Pathol.

[27] Awaji M, Futakuchi M, Heavican T, Iqbal J, Singh RK (2019). Cancer-Associated fibroblasts enhance survival and progression of the aggressive pancreatic Tumor Via FGF-2 and CXCL8. Cancer Microenvironment: Official Journal of the International Cancer Microenvironment Society.

[28] Raja A, Park I, Haq F, Ahn SM (2019). FGF19-FGFR4 signaling in Hepatocellular Carcinoma. Cells.

[29] Hu L, Cong L (2015). Fibroblast growth factor 19 is correlated with an unfavorable prognosis and promotes progression by activating fibroblast growth factor receptor 4 in advanced-stage serous ovarian cancer. Oncol Rep.

[30] Zaid TM, Yeung TL, Thompson MS, Leung CS, Harding T, Co NN, Schmandt RS, Kwan SY, Rodriguez-Aguay C, Lopez-Berestein G (2013). Identification of FGFR4 as a potential therapeutic target for advanced-stage, high-grade serous ovarian cancer. Clin cancer Research: Official J Am Association Cancer Res.

[31] Ghedini GC, Ronca R, Presta M, Giacomini A (2018). Future applications of FGF/FGFR inhibitors in cancer. Expert Rev Anticancer Ther.

[32] Tiong KH, Mah LY, Leong CO (2013). Functional roles of fibroblast growth factor receptors (FGFRs) signaling in human cancers. Apoptosis: An International Journal on Programmed cell Death.

