# Supplementary figures and images for: The anti-tumor effects of AZD4547 on ovarian cancer cells: differential responses based on c-Met and FGF19/FGFR4 expression
Source: Cancer Cell Int. 2024 Jan 25;24:43. doi: 10.1186/s12935-024-03235-2 (PMC10811874; doi:10.1186/s12935-024-03235-2)

**Fig. 1**

**A**

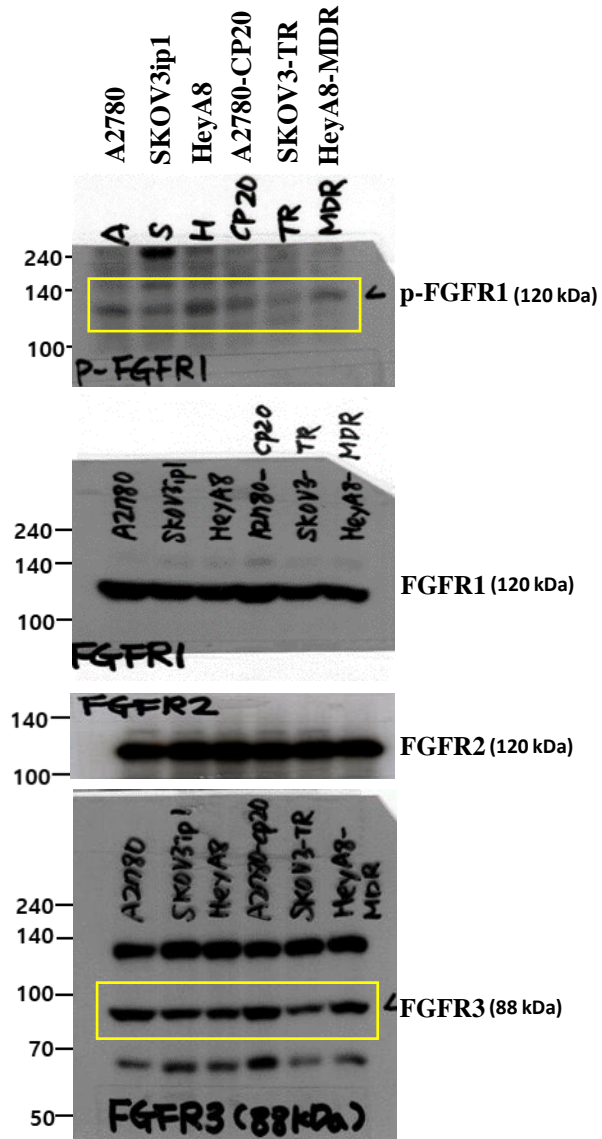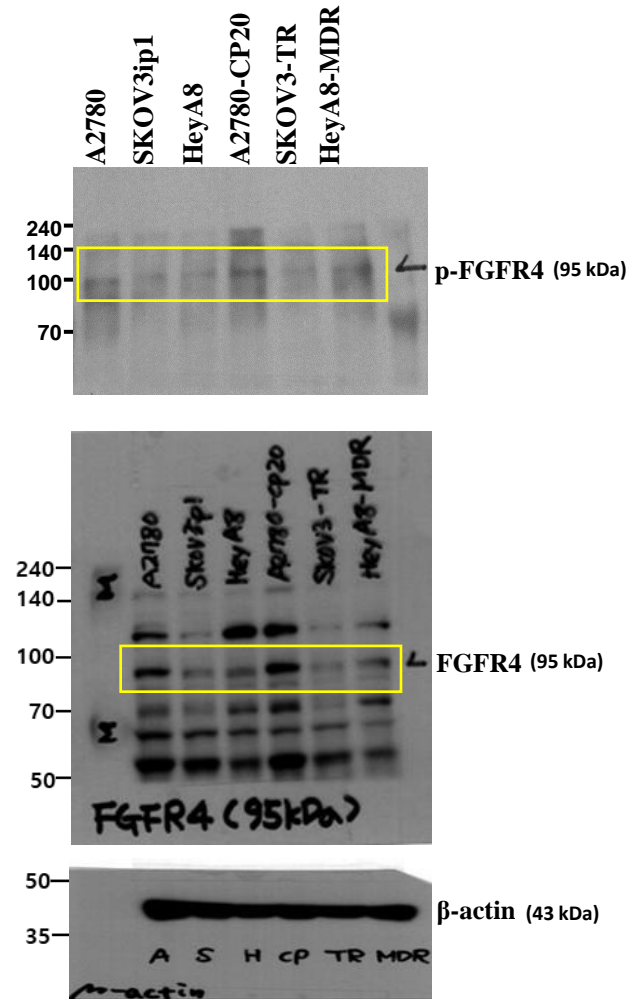

**Fig. 4**

**A2780**

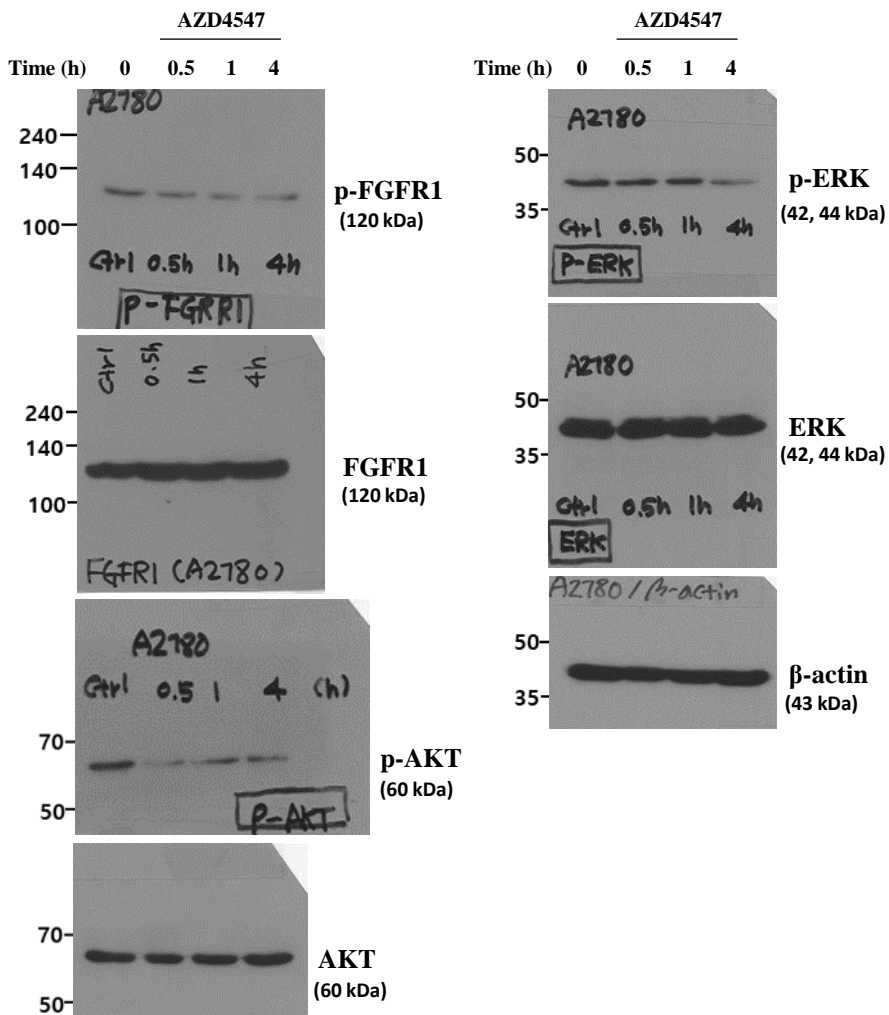

**SKOV3ip1**

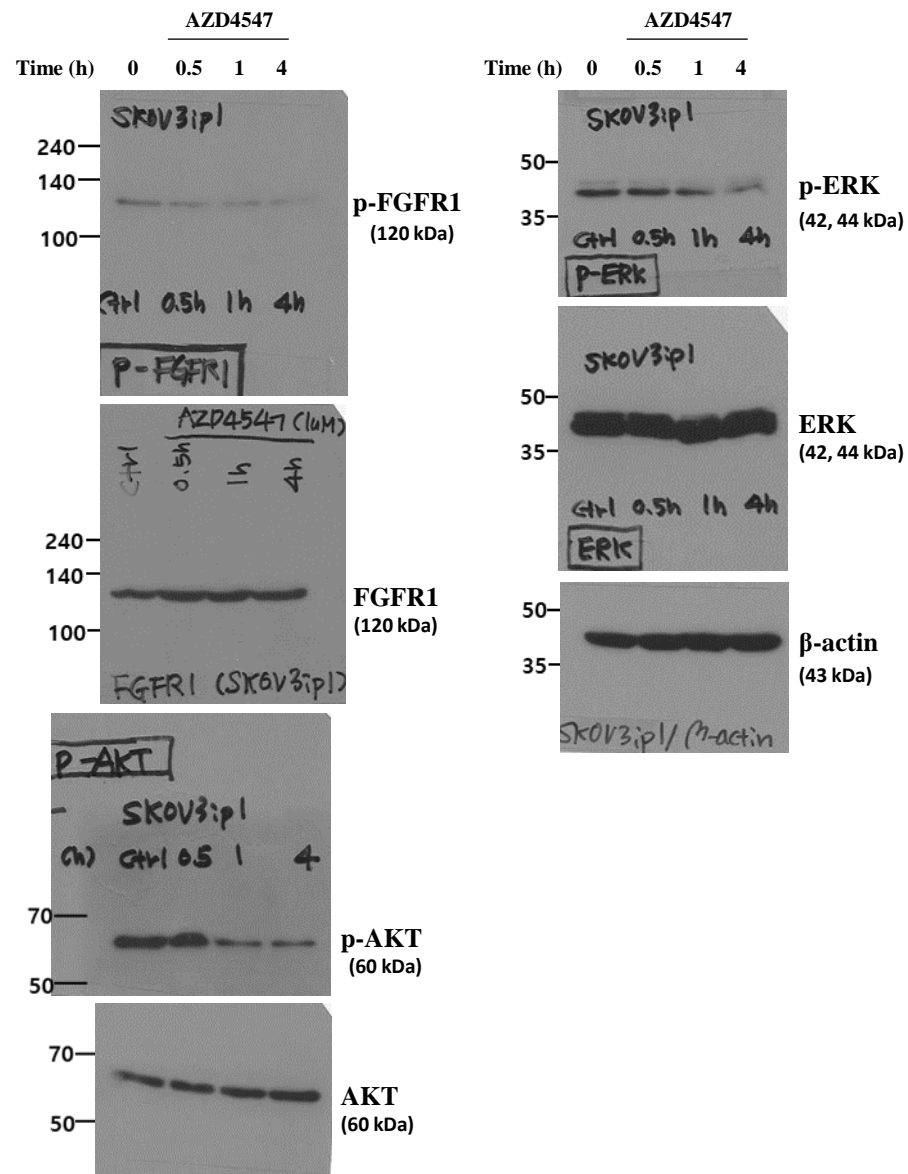

**Fig. 4**

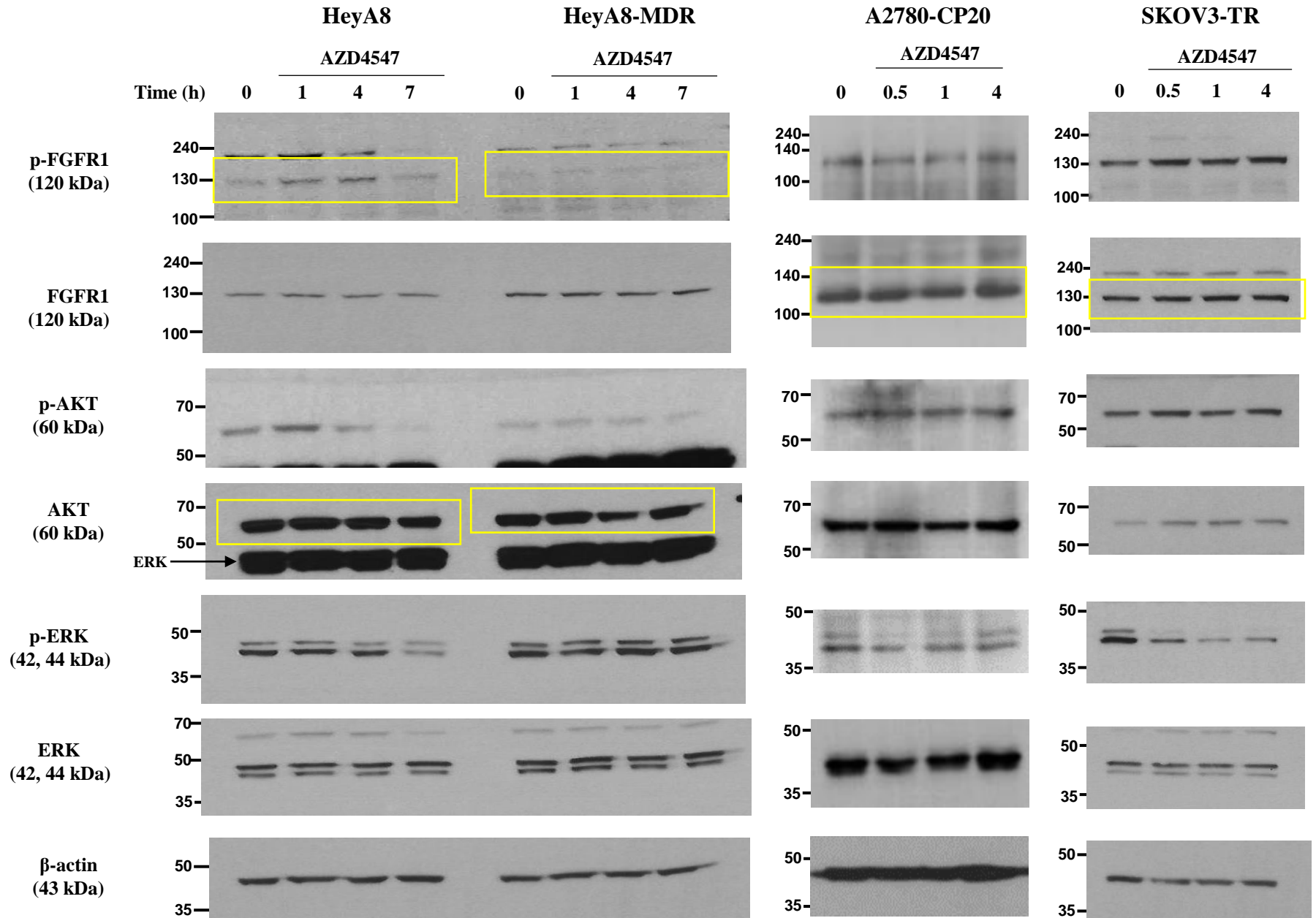

Fig. 4

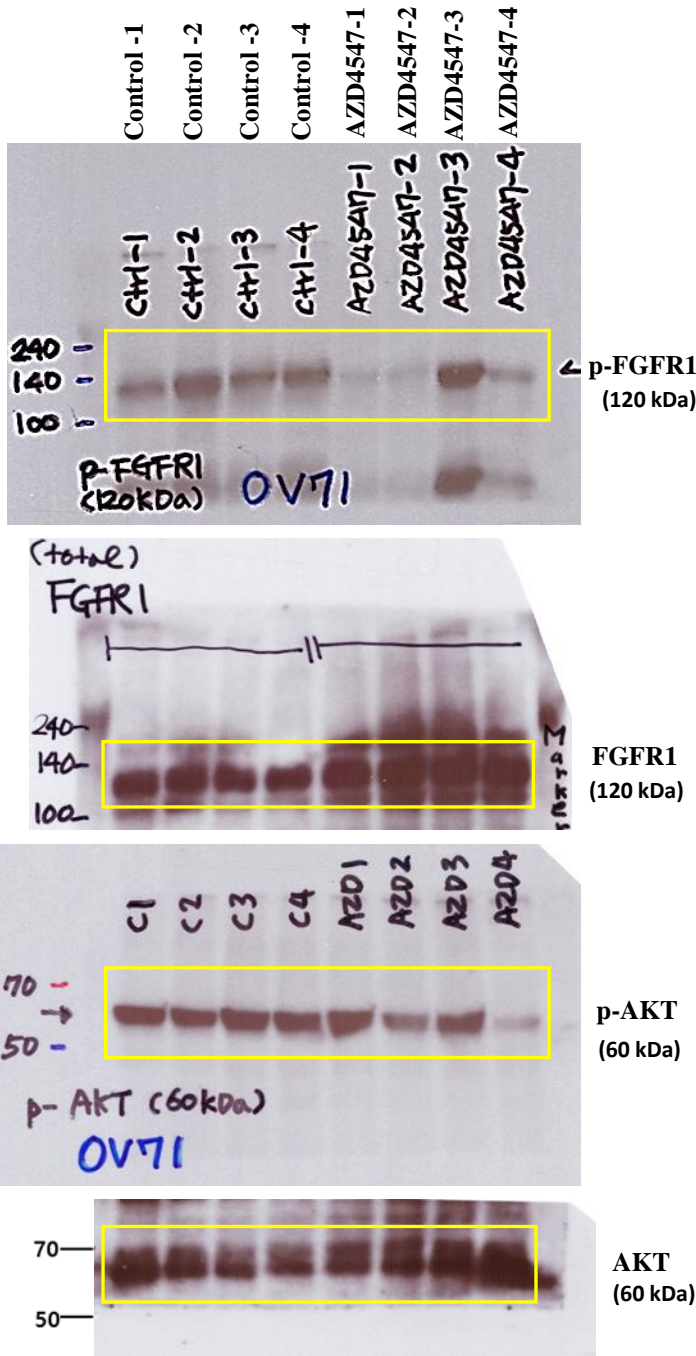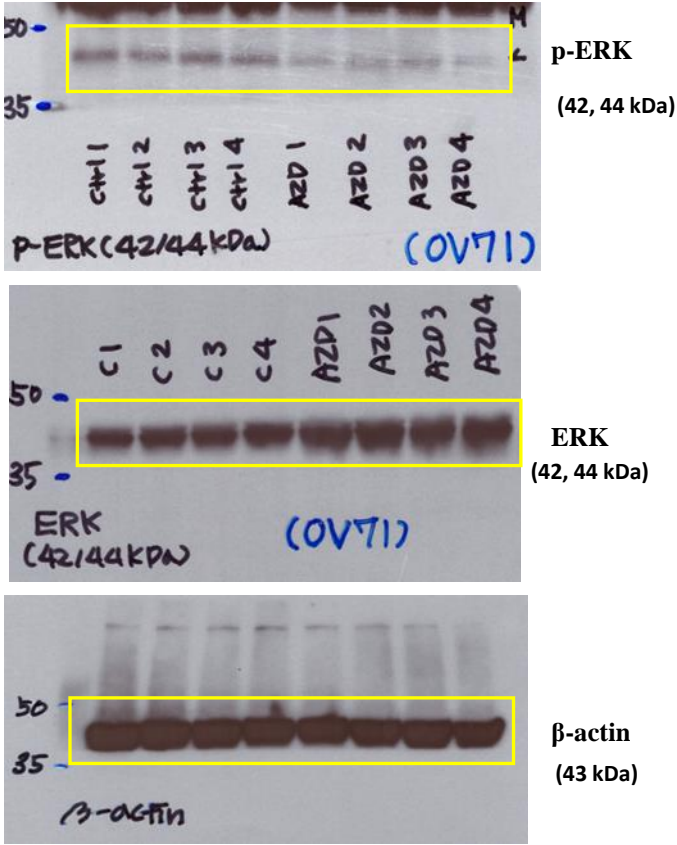

**Fig. 5**

**B**

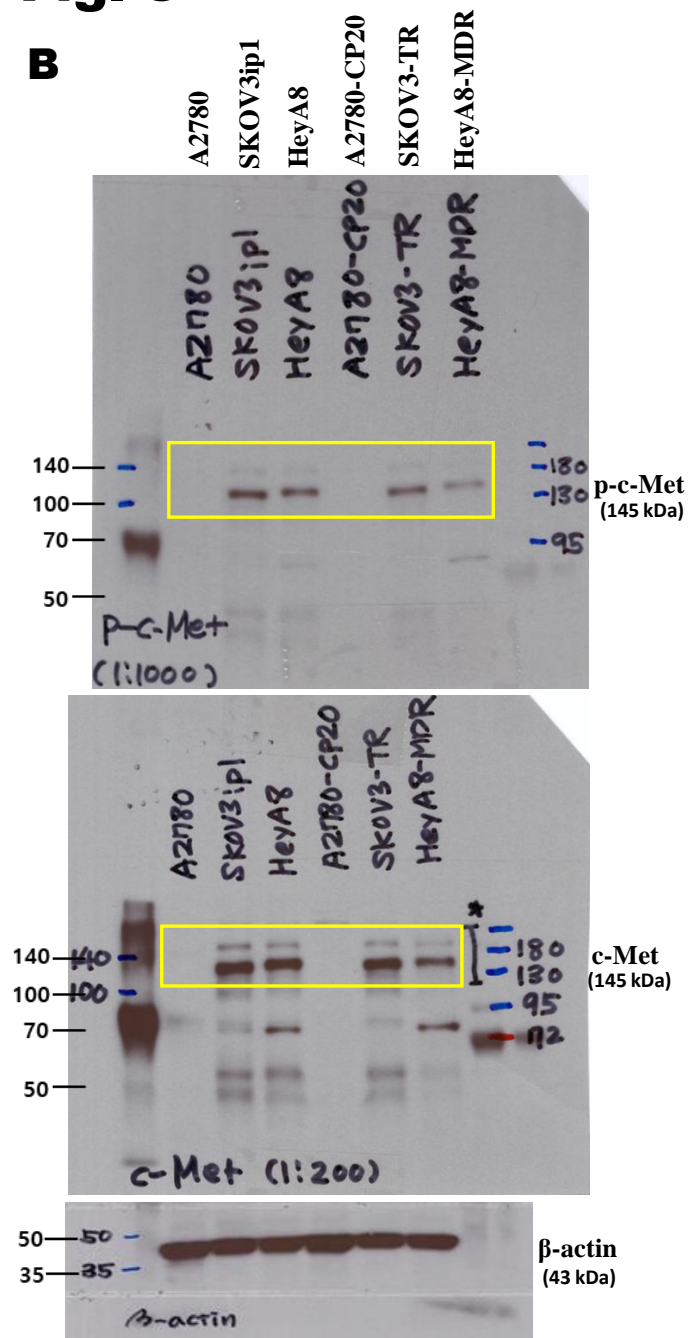

**D**

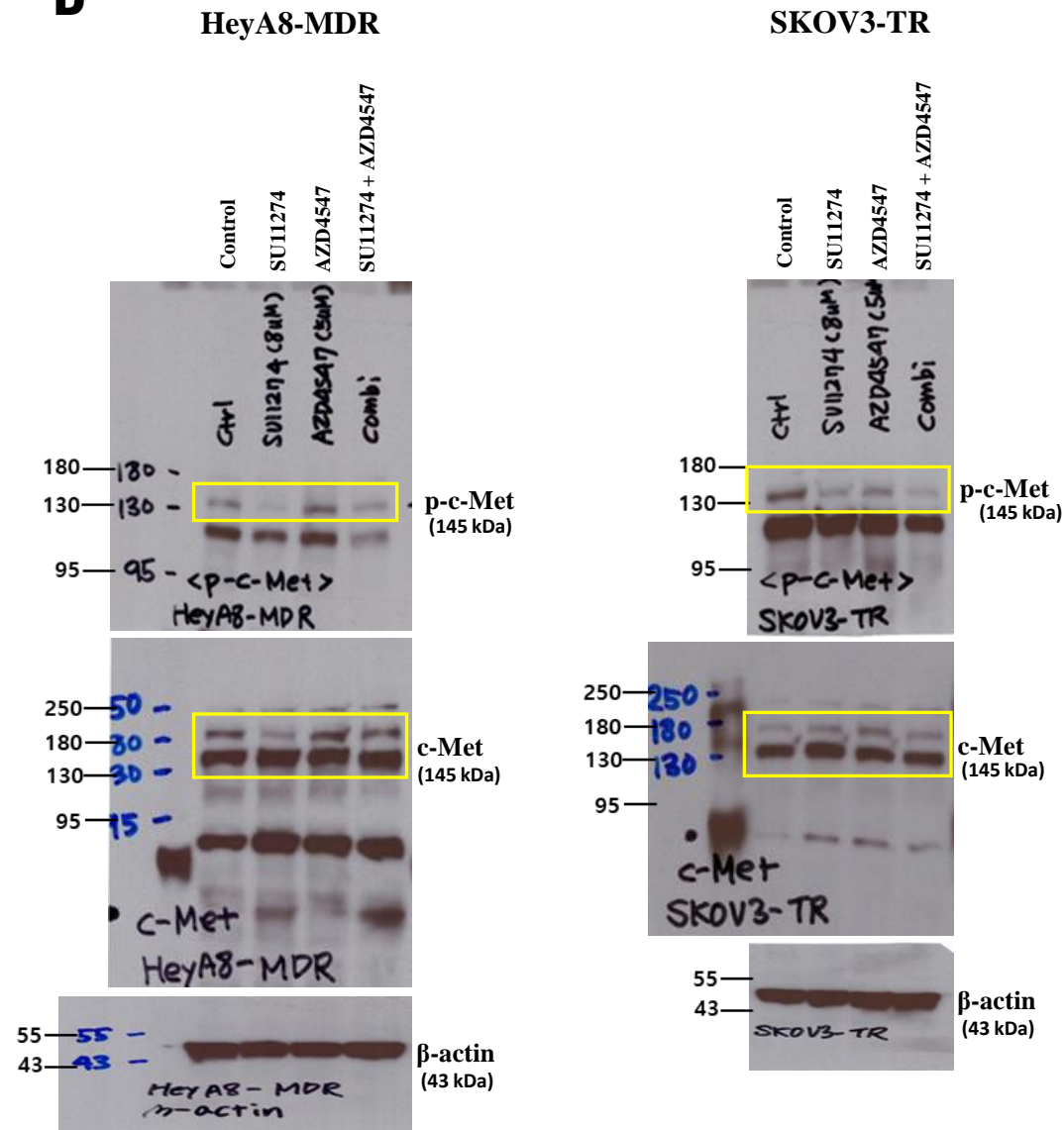

Supplement: Supplementary file 1 — Supplementary Material 1 [file 12935_2024_3235_MOESM1_ESM.pdf]
